# A Carotid Body Tumour Mimicking Richter’s Transformation of Chronic Lymphocytic Leukaemia

**DOI:** 10.7759/cureus.24262

**Published:** 2022-04-18

**Authors:** Younus Qamar, Maryam Gulzar, Amna Qamar, Noreen Rasheed, Imran Syed

**Affiliations:** 1 Department of Radiology, Basildon and Thurrock University Hospital, Basildon, GBR; 2 Department of Radiology, University of Liverpool, Liverpool, GBR

**Keywords:** richter’s transformation, shamblin, head and neck tumors, carotid paraganglioma, chemodectoma, lyre sign, fontaine sign, b-cell lymphoma, non‑functional paraganglioma, carotid body tumour

## Abstract

Carotid body tumours (CBT), also called carotid paragangliomas, are highly vascular glomus tumours that originate from paraganglionic cells of the carotid body. They are frequently asymptomatic, insidious, and non-secretory in nature. They typically present as a large, non-tender, pulsatile neck mass. Careful evaluation of a neck mass, with the aid of imaging, is necessary to avoid a misdiagnosis. We herein describe a case of a 77-year-old gentleman with a background of chronic B-cell lymphocytic leukaemia, who was found to have a rapidly enlarging, asymptomatic neck mass along with multiple enlarged lymph nodes in the axillae. Given his past medical history, the preliminary diagnosis was Richter’s transformation. However, the characteristic splaying of the internal and external carotid arteries on imaging prompted us to consider the diagnosis of a CBT. The patient was referred to vascular surgeons for surgical excision of the tumour. Histology confirmed that the neck mass was indeed a CBT. Ultrasound-guided core biopsy of the axillary lymph nodes revealed a concomitant Hodgkin-like Richter’s transformation. This case exemplifies how we were able to differentiate between a CBT and nodal mass with the aid of various imaging modalities. An accurate diagnosis allows clinicians to deliver the appropriate management; the treatment for CBT is surgical excision, whereas chemotherapy is the first-line treatment for Richter’s transformation.

## Introduction

Carotid body tumours (CBT), also called carotid paragangliomas, are highly vascular glomus tumours that originate from paraganglionic cells of the carotid body [1]. They are frequently asymptomatic, insidious, and non-secretory in nature. They typically present as a large, non-tender, pulsatile neck mass between the age of 50 and 70 years and have a female predilection [2]. They are classically located at the common carotid artery (CCA) bifurcation and display characteristic splaying of the internal and external carotid arteries on imaging (Lyre sign) [3]. Careful evaluation of a neck mass, including with the aid of imaging, is necessary to avoid a misdiagnosis. An accurate diagnosis helps to deliver the best possible care to our patients. With CBT, surgical resection is potentially curative and has a favourable prognosis [2]. Herein, we present the case of a 77-year-old gentleman with a background of chronic B-cell lymphocytic leukaemia, who was found to have a rapidly enlarging, asymptomatic neck mass along with multiple enlarged lymph nodes in the axillae. With the help of various imaging modalities, we were able to establish the correct diagnosis and treat the patient accordingly.

## Case presentation

**Figure 1:**
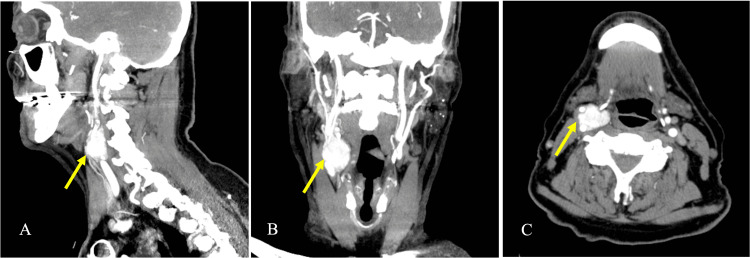
Computed tomography (CT) scan of the neck. Images showing a well-defined and intensely enhancing lesion at the right common carotid artery (CCA) bifurcation, approximately 2.24 cm in craniocaudal dimension, causing splaying of the vessels (Lyre sign). Panel A shows sagittal view. Panel B shows coronal view. Panel C shows axial view.

A 77-year-old Caucasian gentleman, with a history of B-cell chronic lymphocytic leukaemia (CLL), had a whole-body computed tomography (CT) scan prior to commencing chemotherapy. The CT scan showed a large, 2.24 cm lymph node located at the bifurcation of the right CCA with no other supraclavicular lymphadenopathy (Figure 1).

**Figure 2:**
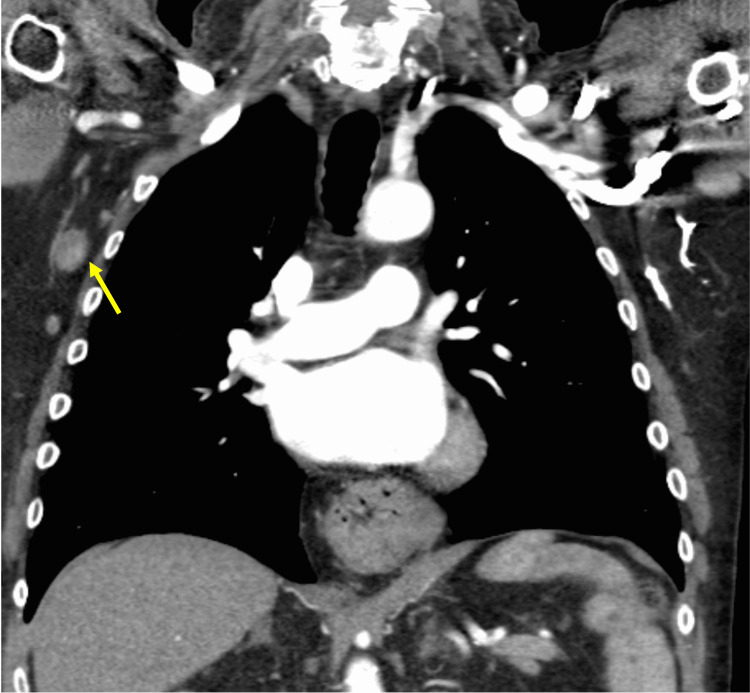
Computed tomography (CT) scan of the chest, in coronal view, showing an enlarged right axillary lymph node.

Additionally, there was an enlarged right axillary lymph node measuring 1.2 cm in diameter (Figure 2).

**Figure 3:**
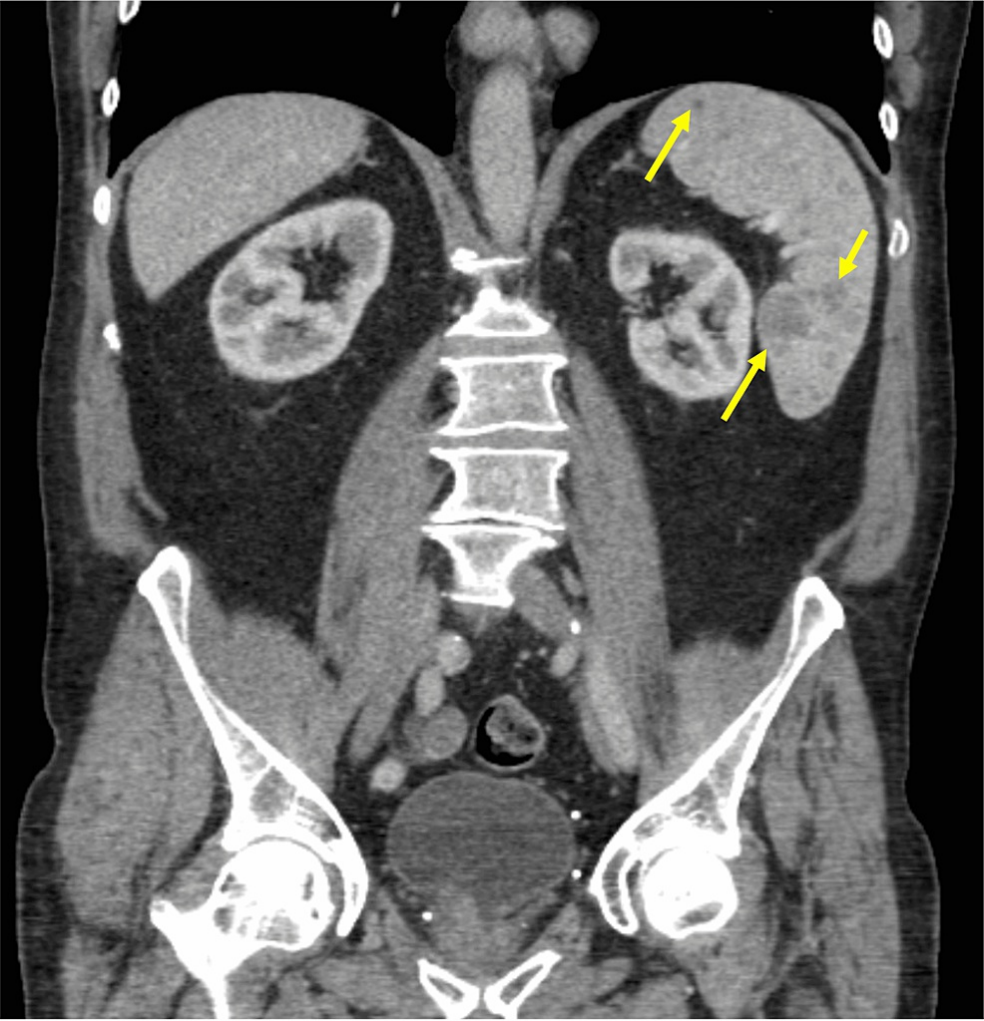
Computed tomography (CT) scan of the abdomen, in coronal view, showing multiple hypoechoic lesions within the spleen.

Furthermore, there were multiple low-density foci present within the spleen (Figure 3).

The patient was investigated for unexplained lymphocytosis approximately 12 years ago (Table 1). He was diagnosed with B-cell CLL on a flow cytometry test, which showed cells typical of B-cell CLL. In the subsequent years, the B-cell CLL behaved indolently, and the lymphocytosis settled. He remained clinically well until 2018, going to the gym regularly. However, since then his general health began to deteriorate and in December 2019, he presented with a history of persistent cough, ongoing for 2-3 months. He denied any typical “B” symptoms of fever, sweating, loss of weight or appetite.

**Table 1:** Full blood count at the time of diagnosis of B-cell CLL. MCV: mean corpuscular volume; CLL: chronic lymphocytic leukaemia

Full Blood Count	
Haemoglobin	136 g/L (130–180)
White cell count	14.1 x 10^9^/L (4.0–11.0)
Platelet count	323 x 10^9^/L (150–400)
Haematocrit	0.40 L/L (0.40–0.52)
MCV	91.3 fL (80–100)
Differential count	
Neutrophil count	3.74 x 10^9^/L (1.7–7.5)
Lymphocyte count	10.5 x 10^9^/L (1.0–4.5)
Monocyte count	0.75 x 10^9^/L (0.2–0.8)
Eosinophils	0.14 x 10^9^/L (0.0–0.4)
Basophils	0.04 x 10^9^/L (0.0–0.1)

**Figure 4:**
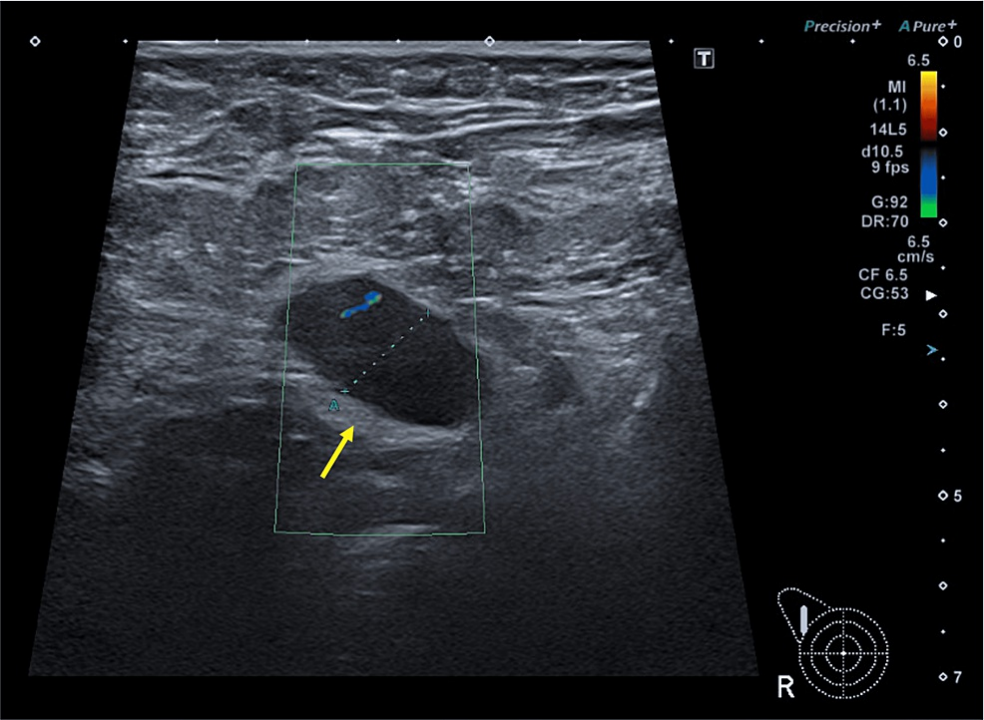
Ultrasound scan (USS) of the right axilla. Scan demonstrating abnormally hypoechoic lymph nodes with loss of central fatty hilum and central vascularity. The short-axis diameter of the largest lymph node within the right axilla measured 12 mm.

Prior to commencing chemotherapy (i.e., at the time the CT scan was performed), the patient was clinically asymptomatic except for a visible, mobile neck swelling noted on the right side with horizontal mobility and restricted vertical mobility (Fontaine sign). The neck swelling had reportedly over a period of 2-3 weeks. A rapidly enlarging lymph node on a background of B-cell CLL was suggestive of Richter’s transformation. Following a discussion in a multidisciplinary team (MDT) meeting, the consensus was to proceed with an ultrasound scan (USS) with possible fine-needle aspiration cytology (FNAC). The USS of the axillae showed a few abnormal, hypoechoic lymph nodes within the right lower axilla. It demonstrated loss of central fatty hilum and central vascularity. The short-axis diameter of the largest lymph node within the right axilla measured 12 mm (Figure 4). In addition, the USS showed a few benign-appearing left axillary lymph nodes.

**Figure 5:**
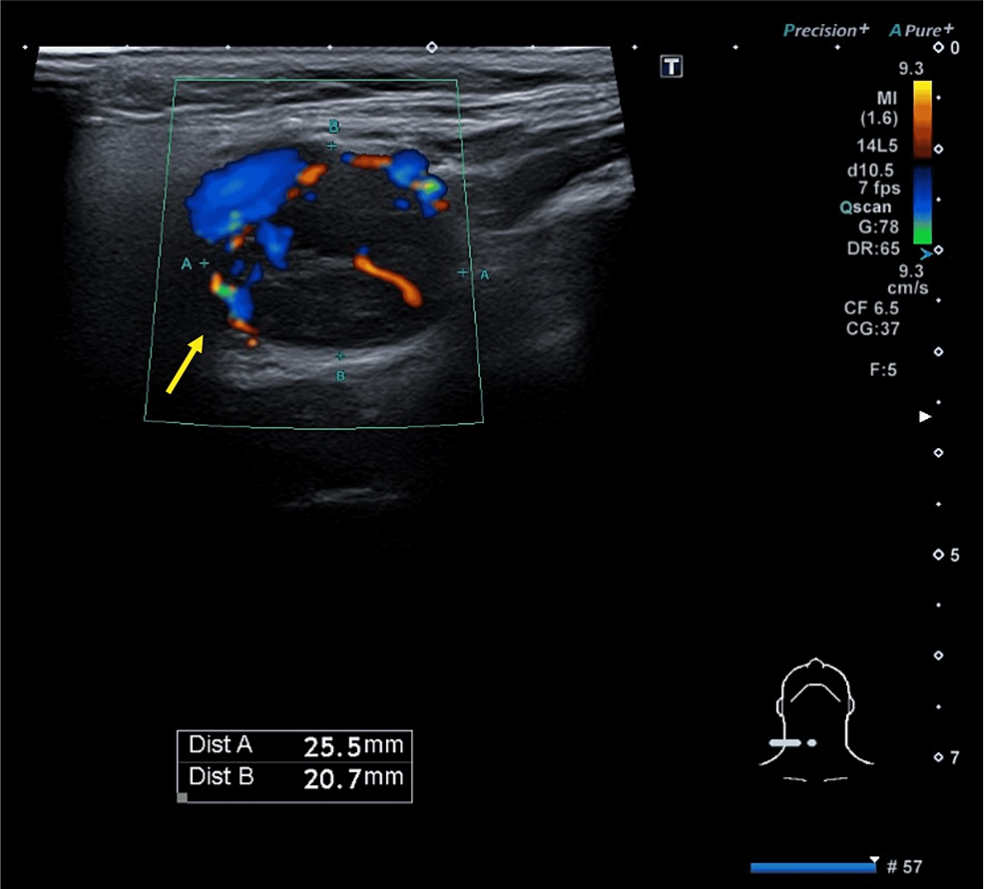
Ultrasound scan (USS) of the neck. Scan demonstrating an oval-shaped, heterogeneously hypoechoic, and highly vascular lesion at the right common carotid bifurcation. The lesion measured approximately 20 x 25 mm.

A USS of the neck demonstrated an oval, heterogeneously hypoechoic, and highly vascular lesion at the right CCA bifurcation. It measured approximately 25 x 20 mm (Figure 5). The appearance of this lesion was consistent with a CBT. To confirm, a contrast-enhanced CT scan of the neck was performed. It showed a well-defined mass splaying the right internal and external carotid arteries (Lyre sign). The mass was not typical of a lymph node, since it enhanced strongly with contrast. It measured 2.0 cm x 2.6 cm x 2.8 cm. Overall, the location and CT appearance of the lesion was in keeping with a CBT. Notably, the patient had no known history of hypertension.

**Figure 6:**
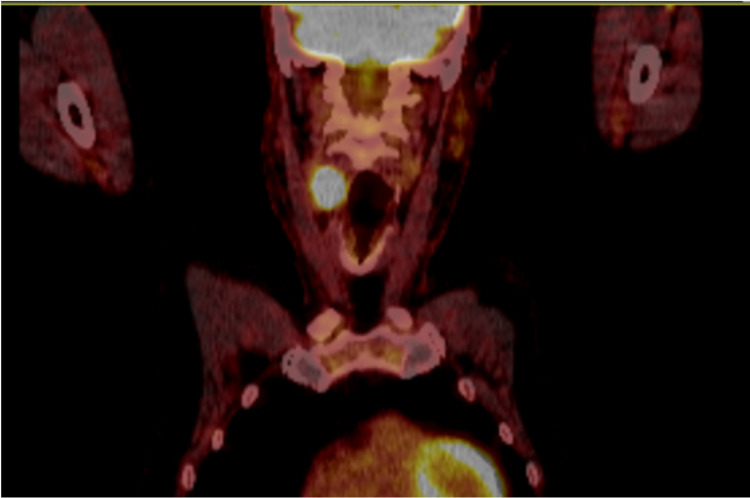
Positron-emission tomography (PET) image in coronal view. Image demonstrating very intense uptake within a soft-tissue abnormality at the right common carotid bifurcation. The maximum standardised uptake value (SUV max) was 29.3.

A positron-emission tomography (PET) CT scan confirmed the diagnosis of a CBT with a very intense uptake seen within the soft-tissue abnormality at the right CCA bifurcation, measuring approximately 2.2 cm x 2.5 cm. The maximum standardised uptake value (SUV max) was 29.3 and the Deauville score was 5 (Figure 6). No other areas of focal fluorodeoxyglucose (FDG) uptake were noted in the rest of the body. The patient was referred to the vascular surgeons for excision of the PET-positive right carotid mass, which was non-secretory in nature; plasma and urinary levels of both metanephrine and normetanephrine (metabolites of adrenaline and noradrenaline, respectively) were within normal ranges. The histology of the right carotid mass definitively confirmed the diagnosis of a CBT, more specifically, a paraganglioma.

A USS-guided core biopsy of the pathologically enlarged right axillary lymph node was performed. The histology confirmed Hodgkin-like Richter’s transformation. Subsequently, the patient was referred for chemotherapy.

## Discussion

CBT, also called chemodectomas or carotid body paragangliomas, are slow-growing and highly vascular glomus tumours that arise from paraganglionic cells of the carotid body [1]. They are classically located at the CCA bifurcation and display a characteristic splaying of the internal and external carotid arteries [3]. They are typically diagnosed between the age of 50 and 70 years and have a female preponderance, just like the other paragangliomas that occur within the head and neck region. It is the most frequently encountered type of paraganglioma in the head and neck region, accounting for approximately 60-70% [2]. They are bilateral in 10% of cases [4]. They are usually benign with <10% that are reportedly malignant in nature [5]. In 7-10% of cases, they belong to an autosomal dominant familial genetic syndrome, such as multiple endocrine neoplasia type 2, neurofibromatosis type 1, von Hippel-Lindau disease (VHL) [6,7].

Richter’s transformation is a term that describes the progression of chronic B-cell lymphocytic leukaemia (CLL) into an aggressive or high-grade non-Hodgkin’s lymphoma (NHL). It reportedly occurs in approximately 5-10% of patients with CLL. Patients usually present with rapidly enlarging lymph nodes, hepatosplenomegaly, and/or elevated serum lactate dehydrogenase (LDH) levels. Non-specific systemic signs include weight loss, night sweats, and fever. Abdominal pain is usually due to an enlarged liver and/or spleen [8]. The nodal disease can occur anywhere in the body; in our patient, USS identified a 1.24 cm right axillary lymph node, which was subsequently biopsied, and the histology confirmed Hodgkin-like Richter’s transformation. Lymph node biopsy is a prerequisite for the diagnosis of Richter’s transformation [8,9].

A non-secretory CBT usually presents as a non-tender, pulsatile neck mass, which is insidious in nature. Patients are usually asymptomatic. It is typically located anterior to the sternocleidomastoid muscle, near the angle of the mandible, and at the level of the hyoid bone [2]. When palpating, a CBT will characteristically mobilise horizontally with restricted movement vertically, owing to its localisation within the carotid sheath; this is referred to as the Fontaine sign [3,4]. Moreover, CBT may implicate any cranial nerve (CN) that runs within the carotid sheath; the glossopharyngeal (CN IX), vagus (CN X), accessory (CN XI), and hypoglossal (CN XII) nerves. Tinnitus, tongue paresis, and dysphagia are symptoms suggestive of CN involvement [10]. Rarely, CBT may secrete hormones, primarily catecholamines such as noradrenaline and adrenaline, however, this is far less common compared to adrenal paragangliomas (pheochromocytomas). Flushing, hypertension, weight loss, nausea, and vomiting are symptoms that should prompt further investigation for a functioning CBT, including assays for noradrenaline, adrenaline and vanillylmandelic acid (VMA). Reportedly, patients with CBT may present with carotid sinus syndrome; symptoms include bradycardia, hypotension, pre-syncope, and loss of consciousness [1,4,5].

In this particular case, the patient had a known diagnosis of chronic B-cell lymphocytic leukaemia. A CT scan identified a rapidly enlarging, asymptomatic lesion in the neck along with multiple enlarged lymph nodes in the axillae. Given his past medical history, the initial impression was Richter’s transformation. However, a PET-CT scan and the characteristic splaying of the internal and external carotid arteries prompted us to reconsider the diagnosis. Eventually, the diagnosis of CBT or paraganglioma was confirmed on histology. US-guided core biopsy of the pathologically enlarged right axillary lymph node confirmed a concomitant Hodgkin-like Richter’s transformation. Therefore, this case exemplifies how we can use various imaging modalities to help differentiate between a CBT and a pathologically enlarged lymph node or nodal mass. This case highlights the importance of doing so, since the management of these two entities differs; a CBT is usually surgically resected, whereas chemotherapy is the first line of treatment for Richter’s transformation.

A contrast-enhanced CT scan characteristically shows a well-defined, rapidly enhancing lesion, causing splaying of the internal and external carotid arteries (Lyre sign). Heterogenous enhancement with contrast is suggestive of haemorrhage within the tumour [3]. On the other hand, if there is clinical suspicion of Richter’s transformation, a CT scan may be performed to look for pathologically enlarged lymph nodes (nodal mass) and/or hepatosplenomegaly. A nodal mass would demonstrate high FDG uptake on a PET-CT scan [9]. Given that a CBT would also appear hypermetabolic (synonymous with high FDG uptake) on a PET-CT scan, it may easily be misdiagnosed as false-positive metastatic lymphadenopathy [10,11]. Clinicians may opt for an octreotide or iodine-123 meta-iodobenzylguanidine (MIBG) single-photon emission computerised tomography (SPECT) CT scan to help differentiate between the two entities if there is a diagnostic dilemma [12]. Moreover, a CBT has a characteristic “salt-and-pepper” appearance on magnetic resonance (MR) imaging; delayed flow or haemorrhage within the tumour represents the “salt” component [13]. However, in clinical practice, the first imaging modality used for investigating a neck swelling or mass is a Doppler USS. With regards to a CBT, it appears as a well-circumscribed, solid, hypoechoic lesion with splaying of the internal and external carotid arteries. With the Doppler effect, we would expect to see a hypervascular lesion [14]. Nevertheless, CT or MR angiography are the mainstay of imaging for the diagnosis of a CBT. Furthermore, it aids surgeons in classifying the CBT according to the Shamblin classification system (Table 2), and in the operative planning. The Shamblin classification system was devised to determine the resectability of a CBT based on the size of the tumour itself and its local spread [11]. A digital subtraction angiography (DSA) helps to identify the dominant artery supplying the CBT and allows for pre-operative embolisation, in the case of large tumours [3,11,14].

**Table 2:** Shamblin classification system. *Group IIIb refers to Group I, II, or IIIa with infiltration to any of the carotid arteries.

Group	Size	Surrounding or infiltrating the carotid arteries	Excision/Resectability
I	< 4 cm	No	Without difficulty
II	> 4 cm	Partially	Difficult
IIIa	> 4 cm	Intimately	Difficult
*IIIb	Any size	Intimately	Requires vascular repair, sacrifice, or vessel replacement, but transmural invasion must be confirmed clinically and/or histologically

The choice of treatment for CBT is determined by several factors, including its location, the spread of the tumour, the patient’s age, and comorbidities. CBT are usually surgically resected and have a favourable prognosis [10,11,14]. In contrast, the first line treatment for Richter’s transformation is chemotherapy, and despite treatment, the prognosis is generally poor with a mean survival period of 5-8 months [8]. Nevertheless, if clinical suspicion of Richter’s transformation exists, such cases warrant discussion in an MDT. Whereas with CBT, prompt, and accurate assessment with the aid of imaging and consideration for pre-operative embolisation (with large, vascular tumours), can help facilitate surgical resection and an excellent recovery. Pre-operative embolisation should be considered for large, highly vascular CBT [1,2,15]. It works by inducing ischaemia and subsequent infarction/necrosis of the tumour. It can also help to shrink large tumours and reduce intraoperative blood loss, operative time, and associated morbidity. Following embolisation, the tumour is surgically resected, however, if the patient declines surgery or is not a suitable candidate for surgery (e.g., elderly, unresectable tumour, recurrence following previous surgical resection), then radiotherapy is an alternative. It can help achieve excellent local tumour control and provide symptomatic relief [1]. Nonetheless, surgical resection of the tumour is superior, given that it is the only form of treatment with curative potential [2,15].

## Conclusions

In the case described above, the patient had a history of chronic B-cell lymphocytic leukaemia and was noted to have an enlarged, asymptomatic neck pass on a CT scan. It was initially misdiagnosed as a malignant cervical lymph node. However, with the aid of imaging and histology, the diagnosis of a CBT or carotid paraganglioma could be established. This is a unique case, in which the patient was also noted to have a pathologically enlarged right axillary lymph node. A USS-guided core biopsy revealed a concomitant diagnosis of a Hodgkin-like Richter’s transformation. Therefore, this case exemplifies how we can use various imaging modalities to help differentiate between a CBT and a pathologically enlarged lymph node or nodal mass. This case highlights the importance of doing so, since the management of these two entities differs; a CBT is usually surgically resected, whereas chemotherapy is the first line of treatment for Richter’s transformation.
